# Exploring the Unexplored: Identifying Implicit and Indirect Descriptions of Biomedical Terminologies Based on Multifaceted Weighting Combinations

**DOI:** 10.1155/2016/1637580

**Published:** 2016-09-06

**Authors:** Sung-Pil Choi

**Affiliations:** Department of Library and Information Science, Kyonggi University, 154-42 Gwanggyosan-ro, Yeongtong-gu, Suwon-si, Gyeonggi-do, Republic of Korea

## Abstract

In order to achieve relevant scholarly information from the biomedical databases, researchers generally use technical terms as queries such as proteins, genes, diseases, and other biomedical descriptors. However, the technical terms have limits as query terms because there are so many indirect and conceptual expressions denoting them in scientific literatures. Combinatorial weighting schemes are proposed as an initial approach to this problem, which utilize various indexing and weighting methods and their combinations. In the experiments based on the proposed system and previously constructed evaluation collection, this approach showed promising results in that one could continually locate new relevant expressions by combining the proposed weighting schemes. Furthermore, it could be ascertained that the most outperforming binary combinations of the weighting schemes, showing the inherent traits of the weighting schemes, could be complementary to each other and it is possible to find hidden relevant documents based on the proposed methods.

## 1. Introduction

Technical terms connote specific technological concepts concisely and play pivotal roles in presenting and leading major points of arguments in scholarly discourses [1]. Moreover, they are commonly used as search queries in scholarly information retrieval. Not surprisingly, their importance has steered many researchers to interesting and important studies on their life cycles (formation, transformation, and extinction) as well as the automatic approaches to term recognition in texts [2–5].

While the use of standardized technical terms is very common in writing scientific literatures, we frequently witness verbose and descriptive linguistic expressions denoting the meaning of those terms in textual documents rather than using the terms directly.

Table 1 shows that authors and writers frequently tend to express obvious technical concepts by using lengthy descriptions instead of brief technical terms, which can be contributed to a few factors. Firstly, they are apt to describe the technical constituents (e.g., terms and jargons) of their documents more clearly by giving the detailed explanations. In some cases, they think that employing existing standard terms is insufficient to describe their evolved technical concepts that could be partially presented by using them. In addition, even if this case is relatively unusual, some authors do not know the existence of the technical terms representing their verbose expressions [6].

**Figure 1:**
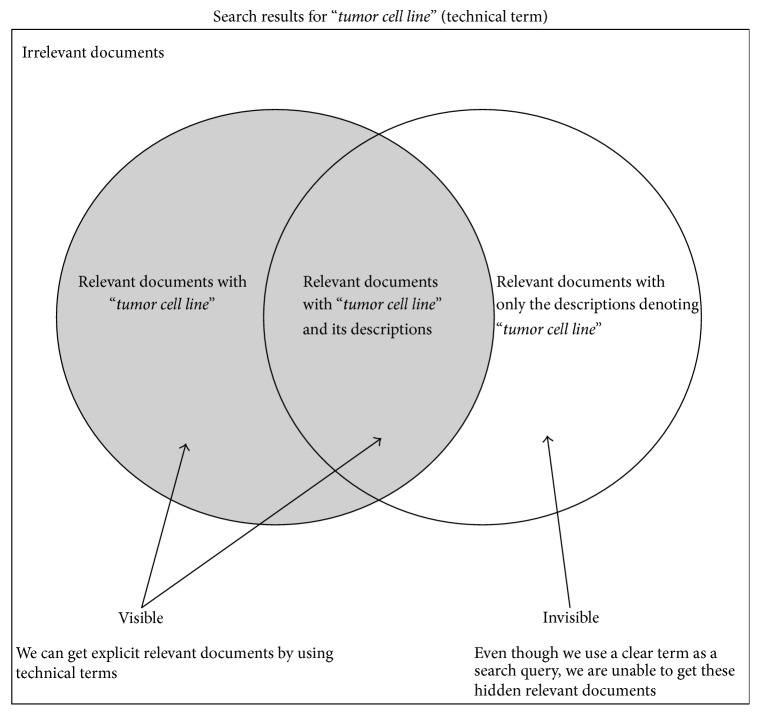
Search results of technical-term-based querying.

The above writing styles introduce some challenges in scholarly information retrieval (IR) [7] and information extraction (IE) from technical documents [8]. In particular, the queries of scholarly document retrieval are mostly technical terms [9] and thus it is very likely to miss the relevant documents not containing the query terms themselves but including only their meanings, which can negatively affect the ranking models by giving implicitly incorrect statistics as shown in Figure 1. Users would overly depend upon an author's keywords and appropriate usages of technical terms without the capability of finding those hidden or varied relevant documents. Conventional IE approaches also cannot detect descriptive named entities such as “…* systems capable of searching relevant texts* … (*text retrieval systems*)” when they apply to technical documents.

Until now, many valuable attempts have been made for detecting important “*concepts*” from textual data by using varied methods [10–13]. However, they mostly aimed to identify or extract just “*important*” chunks or phrases from texts in order to boost the effectiveness of IR or other related text mining tasks. That is to say, they were not interested in textual chunks that possess names or terms of their own in this context. In order to retrieve or analyze scholarly information effectively, however, it is vital to tackle this problem first instead of trying to find important but vague textual expressions. As one of the initial obvious trials to resolve the problem above, Choi and Myaeng [6] introduced a new notion called “*terminological paraphrase* (*TP*)” to indicate textual expressions denoting technical terms. In addition, they developed 6 ranking (in general, the ranking model involves the computation of index (term and PAT) weights as well as the similarities between the two textual chunk vectors; refer to [6] for more details) models to retrieve and extract them from biomedical abstracts.

This paper introduces an enhanced TP extraction system in which multiple term and PAT (predicate argument tuple) weighting schemes can be complementarily combined to extract more hidden TPs from texts. Based on the previously proposed 6 weighting schemes in [6], the system can apply multiple weighting schemes simultaneously to the TP extraction procedure for more achievement of the diversity of the extracted terminological paraphrases. In other words, given that each scheme could extract different TPs from others, it is reasonable to assume that their combinations could extract more diversified TPs without harming the accuracy of the extraction results [10]. A series of intensive experiments is conducted to evaluate the performance of the weighting combinations and check the assumption above. In particular, in the course of finding the optimal binary combination showing the best accuracy, the experimental results and their analysis reveal the complementary characteristics of all the weighting schemes in the combinations. This could lead to more advanced approaches with more novel weighting schemes and further combinations (e.g., ternary, quaternary, etc.).

The rest of the paper is organized as follows.  Section 2 discusses important related works. There is an explanation about the architecture of the system implemented in this paper and the six weighting schemes used by the system in Section 3.  Section 4 presents the experimental results using the proposed system and the evaluation collection by showing the results of the parameter tuning of the two weighting schemes as well as the performance of the combinatorial approaches proposed in this paper. In addition, Section 5 briefly analyzes the experimental results and highlights the research contributions of this paper. In the conclusion, future research is suggested, as well as summarizing the significance of this study.

## 2. Related Works

The research purpose of this study is to expand the coverage of scholarly information access by inspecting the conceptual expressions of terms hidden in texts; thus, one of the most closely connected research areas is “*query expansion* (QE) [9, 11].” In particular, the “*automatic term mapping*” method [11] was introduced in which user queries are connected to MeSH's descriptors, journal titles, or author names in an autonomous manner by analyzing their profiles and log information. However, the approach expands queries by using synonyms in MeSH and hence cannot identify descriptive expressions of either a user's queries or terms apart from the fact that considerable mistakes occur in mapping queries to the above internal resources. The need of controlled vocabularies of fine quality in query expansions makes many researchers focus more on “*pseudo relevance feedback* (PRF) [12, 13]” to expand the search coverage effectively to users' intention. As noticed in [11], however, both QE and PRF do not contribute much to the enhancement of scholarly document retrieval. There was also an attempt to extract various expressions denoting technical terms from the bug reports of software [14]. While that research suggested possible approaches to the identification of terminological paraphrases for the first time, they set limits to mainly noun phrases, which makes their work virtually identical to the synonym extraction [15] or other term-based QE methods.

There has been another research area called “*biomedical concept recognition *[16–18].” Its purpose is to recognize biomedical terms from ontologies in texts, which remains a very open research problem. The only difference with the conventional terminology recognition problems is that the recognized and extracted terms are elements of some existing ontologies. On the other hand, the aim of this paper is to extract linguistic expressions consisting of various multiple words and phrases denoting the concepts of target terms.

**Figure 2:**
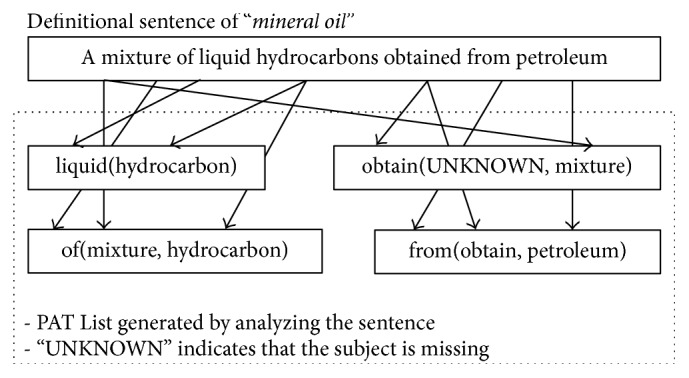
Predicate argument tuples (PATs) from a sentence.

As aforementioned, another line of research tried to identify various types of linguistic expressions apart from noun phrases by converting term definitions as well as all the target texts into predicate argument tuples (PATs) [6]. A PAT can be defined as a syntactical unit of predicate argument structure (PAS) [19] that consists of one or two arguments and a grammatical relation (a.k.a.* predicate*) as shown in Figure 2.

With all the sentences of a target database and the definitions of a set of technical terms converted as above, one approach was to extract syntactical variants of the definitional sentences from the database by matching the PATs [6]. Despite the successful outcomes, this approach was insufficient to extract more dissimilar sentences of the identical meanings of the target terms. To overcome this limitation, this paper extends the method by incorporating multiple conversion models in addition to the PAT-based approach.

## 3. Methods

**Figure 3:**
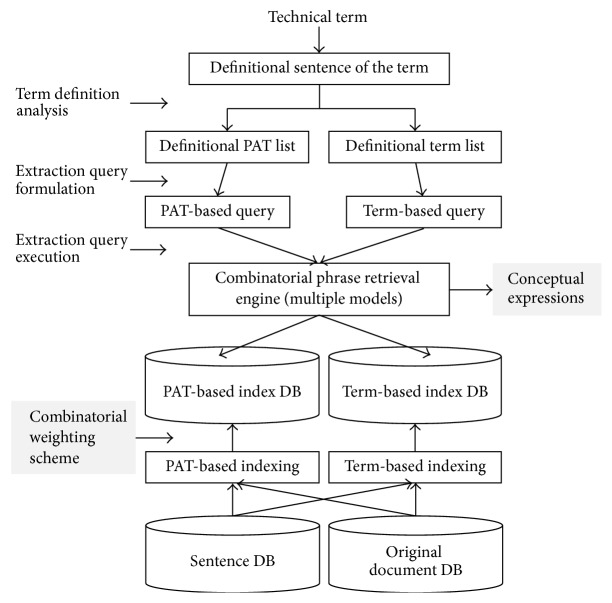
Process of extracting conceptual expressions of a technical term from databases.

This section presents the proposed approaches with the entire architecture of this system which exploits and expands the conventional information retrieval mechanisms, such as inverted files, query processing, and multiple retrieval models, as shown in Figure 3.

### 3.1. Term Paraphrase Extraction Procedure

Figure 3 shows the entire process of this system for extracting conceptual expressions of input technical terms. As aforementioned, the system is analogous to information retrieval engines in that it involves query processing, searching, indexing, and so forth, which possibly speed up the whole extraction process. Firstly, with an input term, its definitional sentences are retrieved from a repository where various term definitions are stored and managed dynamically. The fetched sentences are syntactically analyzed and manipulated to produce a set of definitional PATs and important terms, which are used as two kinds of queries (PAT-based query and term-based query) for finding analogous phrases in the database. The combinatorial phrase search engine is equipped with basically six retrieval models that will be explained later. In particular, the engine can combine multiple models to hopefully expand the search coverage without degrading precision.

**Figure 4:**
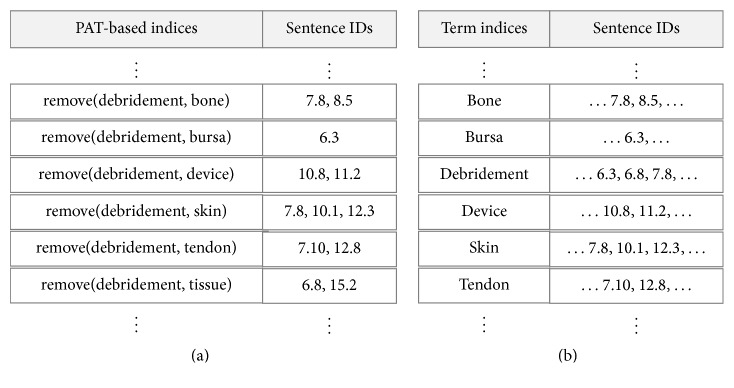
PAT-based index file (a) and term-based index file (b).

For a PAT-based phrase retrieval, one advance was introducing a special inverted file structure in which all the keys are PATs associated with appropriate identifiers of the sentences having the PATs [6]. In this study that advance was revised by reinforcing a word-based indexing scheme to facilitate the combined search mentioned before, as seen in the right side of Figure 4.

For the sake of brevity, there are omissions of additional statistical information such as total term frequency or sentence frequency for computing term significance in the figure. One also notes that all the sentence identifiers are composed of document numbers and the positions in the documents. With the above indexing schemes, as well as various searching methods, the system finds the most analogous sentences or phrases with the input definitional sentences from the database.

### 3.2. Multiple Weighting Schemes and Their Combinations

As proposed in [6], this section briefly explains totally the four weighting schemes to be used in the relevance computation when this system extracts or retrieves conceptual expressions with respect to input query terms. As a weighting scheme computes the significance of search elements (e.g., terms and phrases), this system should consider two kinds of those elements (terms and PATs) together in the process. Firstly, two weighting schemes are used, based both on* binary *(*w*_bin_) and on IDF (inverse document frequency) (*w*_idf_) for a term *t* as follows:

(1) wbint=0,if t is absent in sentence,1,otherwise.widft=log⁡ND−d∈D:t∈d+0.5d∈D:t∈d+0.5,

where *D* is the whole set of documents and *N*_*D*_ is the total number of them in the collection.

Because local weighting schemes, such as TF*∗*IDF, are known to be statistically unreliable for sentence or phrase based information retrieval [20], this paper uses only global ones. Also, the significance of a PAT can be computed in the following two ways:

(2) wbinPAT=0,if the PAT is absent in sentence,1,otherwise.widfPAT=widftprdtarg1,…,targn=1−λwidftprd+λ∑iwidftargi=1−λlog⁡ND−d∈D:tprd∈d+0.5d∈D:tprd∈d+0.5+λ∑ilog⁡ND−d∈D:targi∈d+0.5d∈D:targi∈d+0.5,

where PAT is a predicate argument tuple to be evaluated, which consists of a set of arguments (*t*_arg_) as well as a single predicate (*t*_prd_), and *λ* is a control factor.

**Figure 5:**
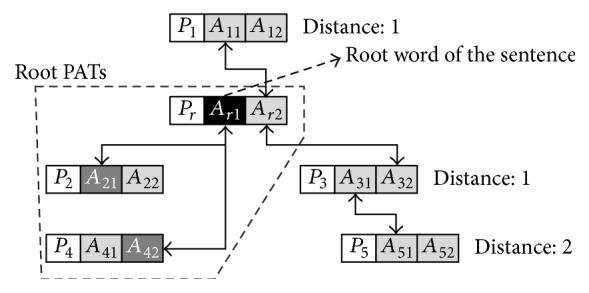
Root PATs and peripheral PATs in a sentence or phrase (note that this figure has been newly introduced in this paper for the better understanding of root and peripheral PATs as proposed in [6].).

In addition, it has been argued that a sentence or phrase has two kinds of PATs, root and peripheral PATs, according to the syntactic roles of their component words, as seen in Figure 5, which should be treated differently [6].

**Figure 6:**
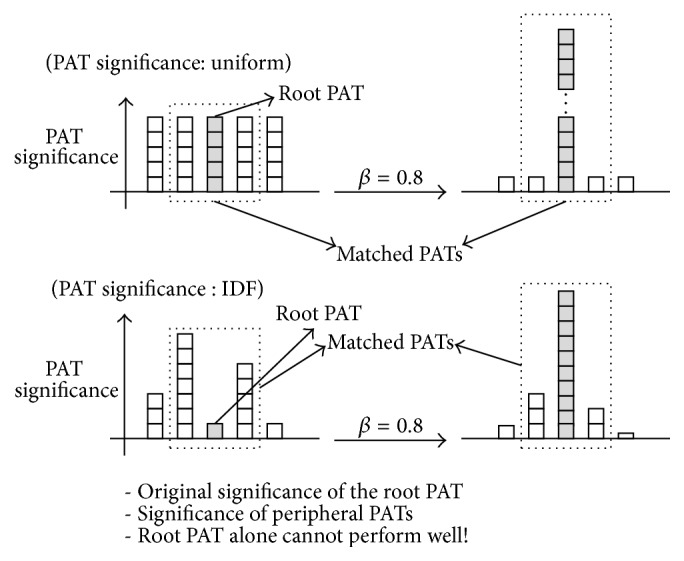
PAT Significance Adjustment (root PAT emphasis process).

A PAT can be a root if and only if it contains the root word of a sentence (verb) or noun phrase (noun). For example, a noun phrase, “*A* ***mixture*** *of liquid hydrocarbons obtained from petroleum,*” has totally four PATs:* liquid *(*hydrocarbon*),* obtain *(UN and* mixture*),* of *(*mixture* and* hydrocarbon*), and* from *(*obtain* and* petroleum*). Because “*mixture*” is the root word,* obtain *(UN and* mixture*) and* of (mixture* and* hydrocarbon*) become the roots while the others are peripheral. It has been argued that, in comparing two sets of PATs, the root PATs of them should be more emphasized and thus readily boosted their significance with the total significance of each set unchanged for better performance [6]. Suppose a sentence *S* contains *n* PATs {*p*_1_,…, *p*_*n*_} and their significances are {*w*(*p*_1_),…, *w*(*p*_*n*_)}. Then adjusted significance of each PAT (*w*_adj_) can be computed as follows:

(3) wadjpi=wpi+β·WS−WrootSWrootS·wpi,if  di=0,wpi2WS−∑pk∈P∧dk=0wadjpk,otherwise,WS=∑pi∈Pwpi,WrootS=∑pi∈P∧di=0wpi,

where *d*_*i*_ is the distance between pi and root PAT, *P* is a set of all the PATs in *S*, and *β* (0 ≤ *β* ≤ 1) determines the boosting degree of root PATs in *S* by yielding some significance of peripheral PATs to them, as shown in Figure 6.

As one can see in Figure 6, (3) takes away weight value from the peripheral PATs and gives it to the root PAT. When one adopts *w*_idf_ as a weighting scheme, each PAT can have distinct significance and therefore the adjustment results in a more radical change in the spread of significance than the uniform scheme (*w*_bin_), as noticed in the bottom of the figure. The adjustment model assumes the following argument: if the root PATs of the two sentences are identical, then it is more likely for the pair to be semantically related to each other.

Based on the above five weighting schemes, one can establish various ranking methods (similarity measures) for sentence or phrase based retrieval by adapting a vector space model as a base frame. In other words, a sentence or phrase can be expressed as a vector of either terms (*w*(*t*_1_),…, *w*(*t*_*n*_)) or PATs (*w*(PAT_1_),…, *w*(PAT_*m*_)) and then one can deduce the similarity of a term definition vector and a sentence vector as follows:

(4) $$\begin{array}{c} \text{sim}\left(\;s,t,w\;\right)=\frac{\sum_{i=1}^{N}w_{i,s}w_{i,t}}{\sqrt{\sum_{i=1}^{N}{\left(w_{i,s}\right)}^{2}}\sqrt{\sum_{i=1}^{N}{\left(w_{i,t}\right)}^{2}}}. \end{array}$$

Here, **s** = {*w*_1,*s*_,…, *w*_*N*,*s*_}, **t** = {*w*_1,*t*_,…, *w*_*N*,*t*_}, and *w* is one of six weighting schemes, which are *w*_bin_(*t*), *w*_idf_(*t*), *w*_bin_(PAT), *w*_idf_(PAT), *w*_adj_(PAT) with *w*_bin_, and *w*_adj_(PAT) with *w*_idf_ aforementioned. Let *W* denote a set of all the weighting schemes and **P** denote its power set. Thus, a proposed combinatorial ranking model can be established as follows:

(5) $$\begin{array}{c} {\text{sim}}_{\text{com}}\left(\;s,t,{Ρ}_{i}\left(\;W\;\right)\;\right)=\underset{\hat{w}\in {Ρ}_{i}\left(W\right)}{max}\left(\;\text{sim}\left(\;s,t,\hat{w}\;\right)\;\right), \end{array}$$

where **Ρ**_*i*_ is an elementary set of the power set of *W* and then (5) simply gives the maximum of the similarity values coming from the input weight schemes denoted by **Ρ**_*i*_. For example, when **Ρ**_*i*_ is {*w*_idf_(*t*), *w*_bin_(PAT), *w*_adj_(PAT)  with  *w*_bin_}, this system computes three similarity values between **s** and **t** based on the weighting schemes and then returns their maximum. This paper combines various ranking models by changing these elementary sets of multiple weighting methods.

## 4. Experimental Results

### 4.1. Experimental Setup

The implementation of the term concept extraction system is based on the process model presented in Figure 3. Automatic indexing was performed by using Enju Parser [19] to extract both PATs and index terms from sentences. As shown in Figure 4, this study developed an inverted file structure embracing those two kinds of indices for the efficient sentence-based retrieval. A couple of the index databases contains various statistical information needed to compute the above six weighting values, as mentioned in Section 3.1.

To evaluate the performance of the proposed weighting combinations, this system used the Terminological Paraphrase Identification (TPI) test collection constructed by [6] (Table 2).

In this collection, each instance has three textual elements (term, definition, and paraphrase) and an annotation (positive, negative, and relative). “*Relative*” means that the meaning of a paraphrase is either broader or narrower than the corresponding term. In the experiments in this study, these “relative” instances were deemed as positive ones in order to strictly investigate the performance of this system. Whereas in another study [6] they only focused on the extraction accuracy of various weighting methods, this paper attempts to seek the optimal ways to combine them for finding more hidden paraphrases.

### 4.2. Parameter Tuning for *w*_adj_(PAT) Scheme

**Figure 7:**
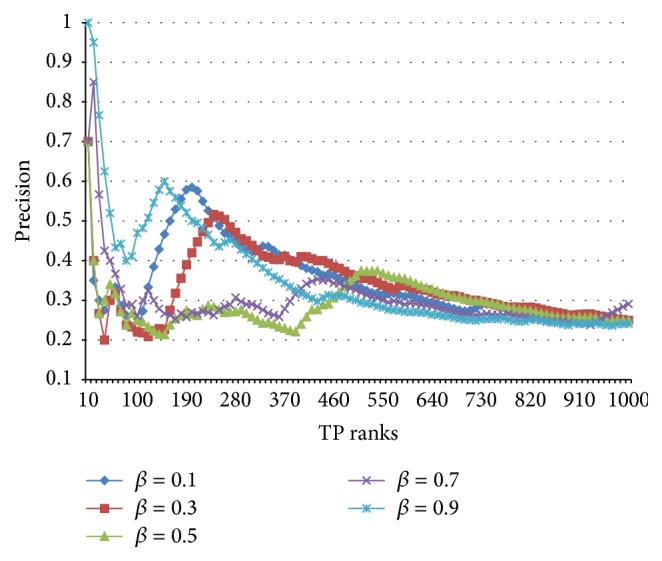
Variance in ranked precisions of “*w*_adj_(PAT) with *w*_bin_” via *β*.

**Figure 8:**
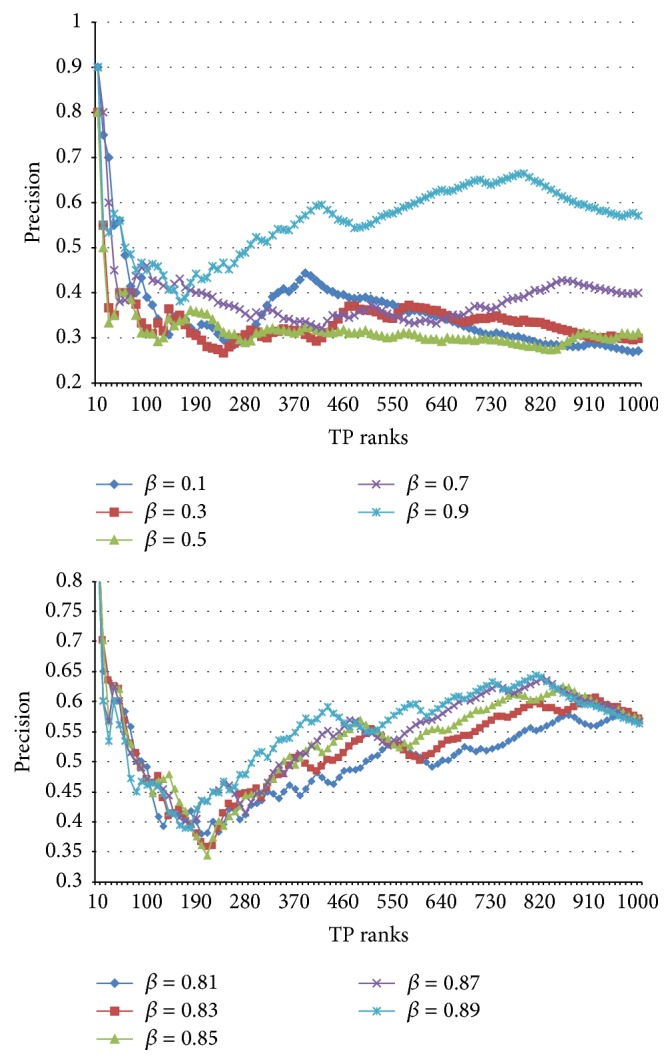
Variance in ranked precisions of “*w*_adj_(PAT) with *w*_idf_” via *β*.

Among all the proposed weighting criterion (*w*_bin_(*t*), *w*_idf_(*t*), *w*_bin_(PAT), *w*_idf_(PAT), “*w*_adj_(PAT) with *w*_bin_,” “*w*_adj_(PAT) with *w*_idf_”) *w*_adj_(PAT) methods have a parameter *β* (0 ≤ *β* ≤ 1) that determines the boosting degree for root PATs in a sentence or phrase, as mentioned in Section 3.2. By using the above TPI collection, in this study an optimal value for *β* was found first. The changes in ranked precision of “*w*_adj_(PAT) with *w*_bin_” and “*w*_adj_(PAT) with *w*_idf_” are shown in Figures 7 and 8, respectively.

As one can see in Figure 7, the parameter *β* does not influence the ranked precision of “*w*_adj_(PAT) with *w*_bin_.” This is because the root PAT emphasis process explained in Section 3.2 had very little impact positively on the entire performance, with no help of the significance of matched PATs. When the significance of every matched PAT is identical (as in the upper side of Figure 6), the root PAT emphasis approach excessively extorts the weight values from the peripheral PATs, which leads to the situation where they can barely participate in the similarity computation.

On the contrary, when an IDF-based PAT significance (“*w*_adj_(PAT) with *w*_idf_”) was used in this study, the changes in the ranked precision of the root PAT emphasis method are very large as one can see in Figure 8. Note that the more the emphasis is upon root PATs by increasing *β*, the better the system shows its performance. In particular, with *β* = 0.9, in other words, when one almost maximally delegates the significance of peripheral PATs to root PATs, the ranked precision reaches an optimal state. Thus, one can draw an analogy from the above results that even though root PATs play key role in finding semantically identical but superficially different expressions of particular phrases, the peripheral PATs are also important factors by compensating the defect of root PAT-centric comparison process. In the following experiments, the best results in this tuning procedure were obtained by using *β* = 0.9 for both “*w*_adj_(PAT) with *w*_bin_” and “*w*_adj_(PAT) with *w*_idf_.”

### 4.3. Duplication Analysis of Weighting Schemes

If all the proposed weighting schemes would extract analogous conceptual expressions, the combinatory approach in this study might be almost useless. Thus, in this section, there is an investigation of the degree of duplications between the six weighting methods before there can be an evaluation of the effect of various combinations. For this part of the study, the following was used: a subset of the TPI collection corresponding to 15 biomedical terms each of which has at least 20 instances in the collection.  Table 3 shows the result of accumulating the number of the rightfully extracted expressions (top 10 ranked items) by incrementally combining the proposed weighting schemes from *w*_bin_(*t*) to *w*_adj_(PAT) with *w*_idf_. The figures in the parentheses, (“(+*n*)”), denote the number of newly extracted indirect expressions by combining additional weighting schemes. For instance, the system only using *w*_bin_(*t*) could extract 10 correct expressions denoting “*Middle Cerebral Artery*” and hence its ranked precision (top 10) is 100%. One can obtain the additional nine expressions by combining *w*_idf_(*t*) to *w*_bin_(*t*), which means the two schemes draw nearly different outcomes and their combination seems to be beneficial in finding hidden expressions corresponding to the term. Also, one can extract a further nine new expressions by repeatedly combining *w*_bin_(PAT) and consequently 28 nonoverlapped, correct expressions out of 30 extracted ones. In this way, with the total combination of the proposed weighting schemes, this system could extract 38 (out of 60) true conceptual expressions of the term whose ranked precision is 63.3%.

Overall, as for the 15 biomedical terms that have 507 indirect expressions in the collection, *w*_bin_(*t*) alone extracted 117 ones (23%) correctly. With *w*_idf_(*t*) combined to this, one can extract 202 expressions (39.8%) and an additional cumulative combination with *w*_bin_(PAT) draws 256 identical phrases (50.5%). Finally, with all the weight schemes combined, one could extract 350 correct expressions out of 507, which means the extraction coverage is over 74%. Furthermore, it is even more worthwhile to note that this study took into account just 10 highly ranked expressions of each method, which shows the beneficial effects of the combinations.

### 4.4. Optimal Weighting Combinations

The experimental results of Section 4.3 show that the 6 weighting schemes have their own characteristics in pinpointing indirect expressions denoting technical terms and it is very likely that their combinations give better diversity and beneficial effects for discovering hidden relevant expressions. Based upon these hypotheses, this study endeavored to find the best binary combination in this section, as shown in Table 4.

**Figure 9:**
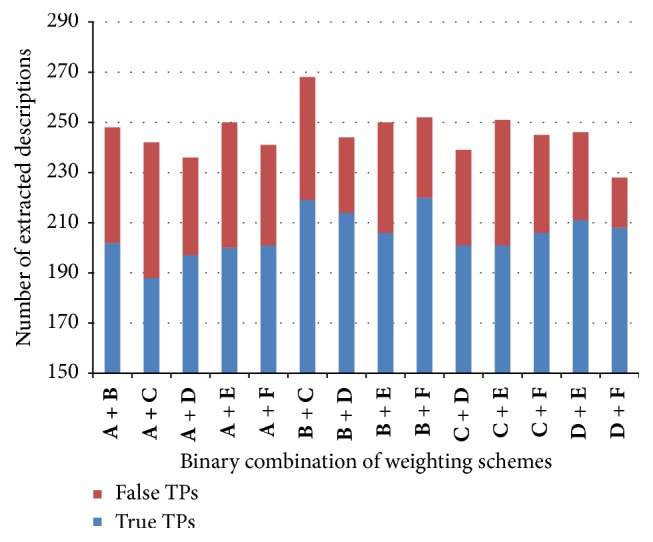
Distribution of true/false descriptions extracted by binary combinations of weighting schemes. (**A**: *w*_bin_(*t*),** B**: *w*_idf_(*t*),** C**: *w*_bin_(PAT),** D**: *w*_idf_(PAT),** E**: “*w*_adj_(PAT) with *w*_bin_,” and** F**: “*w*_adj_(PAT) with *w*_idf_ ”).

Table 4 and Figure 9 show the performance of the 14 binary combinations with presenting the numbers of both correctly and incorrectly extracted expressions as well as their precisions. Note that the two combinations *w*_idf_(*t*) plus “*w*_adj_(PAT) with *w*_idf_” (220) and *w*_idf_(*t*) plus *w*_bin_(PAT) (219) are extracting the largest number of the correct expressions, which could reflect that the IDF-based weighting schemes render beneficial effects to this combination process. In particular, even though the performance of *w*_idf_(*t*) alone was relatively low, as shown in [6], the PAT-based weighting schemes seem to be an efficacious partner in binary combinations. Additionally, *w*_idf_(PAT) plus “*w*_adj_(PAT) with *w*_idf_” outperforms all the other combinations in its precision to extract the smallest number of incorrect expressions (20). From this experiment, one could reasonably conjecture that it is important to apply lexical weighting schemes into both word-based and PAT-based schemes to boost the accuracy of extracting indirect expressions of biomedical terms.

## 5. Discussions

As shown in the experimental results, our combinatorial weighting approach to extract indirect descriptions of biomedical terms could discover additional expression incrementally, which implies that with further weighting schemes used the combinations could find more hidden expressions from scientific terms.  Table 5 shows the results of extracting equivalent expressions of other biomedical terms from PubMed database.

Although some descriptions presented in Table 5 cannot be directly related to their terms due to the ambiguity of the definitions of serval target terms (“*flavor delivery agent*” → “*vanillic acid*”), the proposed system seems to catch many useful variations of the terms such as “redness and cracking of skin” → “dermatitis” and “disorders of cornea” → “corneal diseases.” In this respect, the contribution points of the paper are as follows:

- In order to find hidden, relevant documents having only indirect and descriptive expressions representing technical terms, this paper proposes combinatorial weighting schemes, which utilize various indexing and weighting methods and their combinations.
- Based on the combinatorial weighting concepts, a practical search engine capable of retrieving the verbose expressions of technical terms in scientific texts is used.
- The experiments showed promising results in that one could continually locate new expressions by combining the proposed weighting schemes. Furthermore, one could find the most outperforming binary combinations of the weighting schemes.
- As a result, our methods could pave the systematic ways to retrieve hidden but relevant documents that were unreachable by using conventional, technical term-based search systems in biomedical domains.

## 6. Conclusion and Future Research

This paper introduced novel combinatorial weighting approaches of manipulating the term definitions as well as target databases to effectively find conceptual expressions of technical terms in the biological domains. Although the ultimate research goal of this study is to enhance the performance of scholarly information retrieval, this paper firstly focused more on finding effective extraction settings for the conceptual chunks denoting technical terms with experimental approaches. For these purposes, this study developed an indirect expression retrieval system for biomedical terms equipped with word-based weighting modules as well as PAT-based indexing approaches. Also, this research formalized and implemented the six weighting schemes in this system.

By using the evaluation collection constructed in another salient study [6], this research group conducted a set of intensive experiments to find the optimal states for extracting (retrieving) conceptual expressions hidden in text. The experiments showed promising results in that one could continually locate new expressions by combining the proposed weighting schemes. Furthermore, one could find the most outperforming binary combinations of the weighting schemes (*w*_idf_(*t*) plus “*w*_adj_(PAT) with *w*_idf_” and *w*_idf_(PAT) plus “*w*_adj_(PAT) with *w*_idf_”).

In order to find relevant scholarly information from the scientific databases, in general, one uses technical terms. However, as argued in other research [21], those technical terms have limits as query terms because there are so many indirect and conceptual expressions denoting the terms in texts. Needless to say, we hypothesize that our study is just at an early stage to tackle this challenging problem and we have much research to do in the future.

For future studies, we will endeavor to invent more novel and effective indexing and weighting models to boost the current performance. Semantic information could be applied to enhance the coverage of the PAT-based schemes. At the same time, we will conduct more rigorous experiments to evaluate the effectiveness of our weighting schemes and their combinations. Finally, this experimentation needs to be more extensive, for example, by adopting many technical terms in the biomedical domains as well as in other scientific and scholarly discourses.

## Figures and Tables

**Table 1:** Biomedical terms and their verbose expressions denoting their meanings.

Biomedical terms	Descriptive and indirect expressions in texts
Impulse control disorders	…the failure to resist an impulse, drive, or temptation and to perform an act that is harmful to the person or to others…
Tumor cell line	The present invention relates to the discovery that renewable stem cell lines can be derived from tumor cells and can be cultured in vitro
Alkaloids	…nitrogenous organic base…
Arachidonic acid	…essential and/or highly unsaturated fatty acids… …unsaturated fatty acids essential fatty acids…
Arteritis	…inflammation of coronary arteries…

**Table 2:** Statistics of TPI test collection [6].

	Positive	Negative	Relative
Size	2,388 (9.8%)	19,855 (81.1%)	2,225 (9.1%)
	24,468 (100%)		

**Table 3:** Results of accumulative combinations of proposed weighting schemes (**A**: *w*_bin_(*t*), **B**: *w*_idf_(*t*), **C**: *w*_bin_(PAT), **D**: *w*_idf_(PAT), **E**: “*w*_adj_(PAT) with *w*_bin_,” and **F**: “*w*_adj_(PAT) with *w*_idf_”).

Biomedical terms (15 terms having more than 20 indirect expressions in the collection)	Number of entire indirect expressions (*T*)	Cumulative results by combining each weighting scheme step by step (top 10)						Total precision (ALL/*T*)
		**A** (max. 10)	**A** + **B** (max. 20)	**A** + **B** + **C** (max. 30)	**A** + **B** + **C** + **D** (max. 40)	**A** + **B** + **C** + **D** + **E** (max. 50)	**ALL** (max. 60)	
Bronchitis, chronic	66	10	17 (+7)	26 (+9)	32 (+6)	39 (+7)	43 (+4)	0.6515
Middle cerebral artery	60	10	19 (+9)	28 (+9)	32 (+4)	34 (+2)	38 (+4)	0.6333
Erythrocytes	54	10	16 (+6)	19 (+3)	19 (+0)	26 (+7)	32 (+6)	0.5926
Leukocytes	49	10	15 (+5)	19 (+4)	24 (+5)	25 (+1)	26 (+1)	0.5306
Hearing loss, bilateral	47	5	9 (+4)	11 (+2)	15 (+4)	15 (+0)	19 (+4)	0.4043
Cellular structures	28	10	15 (+5)	17 (+2)	19 (+2)	24 (+5)	26 (+1)	0.9286
Lymphocytes	27	10	18 (+8)	22 (+4)	25 (+3)	25 (+0)	25 (+0)	0.9259
Immune sera	26	9	13 (+4)	17 (+4)	19 (+2)	23 (+4)	23 (+0)	0.8846
Neoplasms	25	2	6 (+4)	6 (+0)	14 (+8)	14 (+0)	14 (+0)	0.5600
Pain, postoperative	24	5	13 (+8)	18 (+5)	20 (+2)	21 (+1)	21 (+0)	0.8750
*Neisseria meningitidis *	21	4	6 (+2)	7 (+1)	7 (+0)	7 (+0)	7 (+0)	0.3333
Disease attributes	20	7	12 (+5)	16 (+4)	17 (+1)	18 (+1)	18 (+0)	0.9000
Lung neoplasms	20	7	12 (+5)	15 (+3)	18 (+3)	19 (+1)	20 (+1)	1.0000
Meningeal arteries	20	8	14 (+6)	17 (+3)	17 (+0)	17 (+0)	18 (+1)	0.9000
Parasympathetic nervous system	20	10	17 (+7)	18 (+1)	19 (+1)	20 (+1)	20 (+0)	1.0000

*Total*	*507*	*117* *(0.2308)*	*202 (+85) (0.3984)*	*256 (+54) (0.5049)*	*297 (+41) (0.5858)*	*327 (+30) (0.6450)*	*350 (+23) (0.6903)*	*0.7413*

**Table 4:** Accuracy of binary combinations of weighting schemes.

Binary combinations of weighting schemes	Performance of the 15 terms		
	Number of true exp.	Number of false exp.	Precision
*w*_bin_(*t*) + *w*_idf_(*t*)	202	46	0.8094
*w*_bin_(*t*) + *w*_bin_(PAT)	188	54	0.7705
*w*_bin_(*t*) + *w*_idf_(PAT)	197	39	0.8321
*w*_bin_(*t*) + “*w*_adj_(PAT) with *w*_bin_”	200	50	0.7865
*w*_bin_(*t*) + “*w*_adj_(PAT) with *w*_idf_”	201	40	0.8302
*w*_idf_(*t*) + *w*_bin_(PAT)	219	49	0.8151
*w*_idf_(*t*) + *w*_idf_(PAT)	214	30	0.8736
*w*_idf_(*t*) + “*w*_adj_(PAT) with *w*_bin_”	206	44	0.8206
*w*_idf_(*t*) + “*w*_adj_(PAT) with *w*_idf_”	***220***	32	0.8738
*w*_bin_(PAT) + *w*_idf_(PAT)	201	38	0.8392
*w*_bin_(PAT) + “*w*_adj_(PAT) with *w*_bin_”	201	50	0.7895
*w*_bin_(PAT) + “*w*_adj_(PAT) with *w*_idf_”	206	39	0.8322
*w*_idf_(PAT) + “*w*_adj_(PAT) with *w*_bin_”	211	35	0.8500
*w*_idf_(PAT) + “*w*_adj_(PAT) with *w*_idf_”	208	***20***	***0.8939***

**Table 5:** Biomedical terms and their extracted descriptions by the proposed system.

Biomedical terms	Term definitions	Extracted term descriptions from biomedical abstracts
Corneal diseases	Diseases of the cornea	(i) Disorders of cornea

Dermatitis	Any inflammation of the skin	(i) Inflammation in skin (ii) Redness and cracking of skin (iii) Redness and swelling of skin (iv) Redness in skin (v) Redness of skin (vi) Redness or inflammation of mammalian skin

Griseofulvin	An antifungal antibiotic	(i) Antiviral antibiotics (ii) Antibiotics antifungal antiviral

Hydrofluoric acid	Hydrofluoric acid	(i) Acid is hydrochloric (ii) Acid which is hydrochloric

Magnesium chloride	Magnesium chloride	(i) mg chloride (ii) mg sodium chloride (iii) mg sodium chloride between about 28.5 mg and about 31.5 mg potassium chloride between about 30.0 and about 36.0 mg calcium chloride

Vanillic acid	A flavoring agent	(i) Flavor delivery agent

Chondrogenesis	The formation of cartilage	(i) Formation of cartilage and/or bone (ii) Formation of new bone

Nervous system diseases	Diseases of the central and peripheral nervous system	(i) Disorders of central and peripheral nervous system
